# Factors impacting implementation of screening, brief intervention, and referral to treatment (SBIRT) programs

**DOI:** 10.1186/1940-0640-8-S1-A73

**Published:** 2013-09-04

**Authors:** Manu Singh

**Affiliations:** 1 JBS International, Inc., North Bethesda, MD, USA

## Introduction

Screening, brief intervention, and referral to treatment (SBIRT) has been identified as an effective public health approach for identifying and treating individuals who use alcohol and/or other drugs at risky levels. Between 2004 and 2009, the Substance Abuse and Mental Health Services Administration (SAMHSA) funded three cohorts of grantees to implement SBIRT services in selected sites. As part of this effort, a cross-site evaluation of SAMHSA’s SBIRT initiative was conducted on the first cohort of grantees and another is currently ongoing for Cohort III to study the process, outcome and economic impact of implementing SBIRT, as well as on the outcomes of patients who receive SBIRT services. The objective of the current paper is to use the select data collected from the SAMHSA-CSAT’s cross site evaluation of the Cohort III grantees and examine the barriers and facilitators encountered by the grantees as they implement SBIRT service delivery.

## Methods

The data sources include: 1) a practitioner survey, and 2) semi-structured interviews with providers, administrators, key stakeholders, and local evaluators. A qualitative analysis was conducted with the interview data to extract themes related to barriers and facilitators in the responses by the grantee, by performance site type, and by the following topics of interest: impact on systems, integration, patient characteristics, referral process, and sustainability.

## Results

Emerging themes will be defined and discussed in relation to the literature and findings from the SAMHSA cross-site evaluation of Cohort I.

## Conclusions

Based on the findings, recommendations for implementing SBIRT in States or tribal organizations by performance site type (e.g., hospital, emergency department, primary care clinic) will be made.

